# Dual contributions of Xrp1 to genome integrity through the DNA damage response and cell competition

**DOI:** 10.1371/journal.pgen.1012309

**Published:** 2026-09-18

**Authors:** Chaitali Khan, Nasser M. Rusan, Nicholas E. Baker

**Affiliations:** 1 Department of Genetics, Albert Einstein College of Medicine, Bronx, New York, United States of America; 2 Cell and Developmental Biology Center, National Heart, Lung, and Blood Institute, National Institutes of Health, Bethesda, Maryland, United States of America; Shanghai Institute of Organic Chemistry, Chinese Academy of Sciences, CHINA

## Abstract

Model organisms may help understand how p53 suppresses tumorigenesis in mammals. In *Drosophila*, the primary transcriptional target of p53 is the gene encoding the bZip AT-hook protein Xrp1, which is another transcription factor. We report that Xrp1 mediates multiple functions of p53 in the DNA damage response (DDR), contributing to p53-dependent gene transcription and DNA damage-induced apoptosis. In addition to this role as a p53 effector, a p53-independent role for Xrp1 in cell competition has been described. Cell competition can remove cells whose genome has been altered by DNA damage and repair. During cell competition, Xrp1 is induced by RpS12, which acts as a sensor of defective ribosome biogenesis. In irradiated discs, p53-independent RpS12-dependent Xrp1 function began as the DDR came to an end, and was even more prominent if p53 function was reduced. Such p53 inhibition resulted in persistence of DNA damage after irradiation, revealed by γH2Av accumulation. Thus, Xrp1 limited the accumulation of abnormal cells resulting from genotoxicity through both the acute, p53-dependent DDR, and also a later mechanism consistent with cell competition removing cells where DNA repair did not restore the normal genome. Both these processes might contribute to the tumor suppressor function of p53 in mammals.

## Author summary

Accurately maintaining somatic cell genomes helps to prevent cancer. This is accomplished in part by a DNA Damage Response that activates repair genes when DNA damage is detected, and can also kill cells where damage is too extensive. A second mechanism is the selective loss of aneuploid cells even after DNA repair has been completed. The Drosophila transcription factor Xrp1 is expressed in both the DNA Damage Response and in many aneuploid cells. We find that Xrp1 mediates most of the gene expression changes caused by DNA damage, and that both DNA repair and the death of badly damaged cells are less effective without Xrp1. We also extend previous studies of aneuploid cells by showing that Xrp1 function continues to contribute to eliminating cells after DNA damage has been resolved, where Xrp1 is induced by RpS12, a protein that signals decreased ribosomal protein gene dose and initiates cell competition. The synergy between these two processes may offer insight into origins of human cancer. P53, one of the most important human tumor suppressor genes, has both these same functions of marshaling the transcriptional DNA Damage Response as well as stimulating cell competition to remove damaged cells.

## Introduction

Xrp1 is a *Drosophila* transcription factor that was discovered as a p53 target and found to contribute to genome integrity, consistent with a role in the DNA damage response [1,2]. More recently, Xrp1 has been found to control multiple types of cell competition [3–11]. Because cell competition can preserve genome integrity, through the removal of aneuploid cells [12,13], we evaluated the role of Xrp1 in DNA repair and its relationship to cell competition in more detail. We confirmed that Xrp1 executes part of the DNA damage response downstream of p53, and a later p53-independent role consistent with cell competition, and present evidence that the DNA damage response and cell competition synergize to maintain genome integrity.

Genome integrity is important in somatic cells as well as in the germline. Defects in DNA repair are associated with elevated cancer incidences, and defects in mitotic fidelity occur in aging [14–17]. Errors in DNA repair can result in aneuploidy, as found in most cancer cells, and both mouse models and human genetic diseases that predispose to aneuploidy are cancer prone [18–20]. The most important human tumor suppressor, p53, is well known as a transcriptional master regulator of the DNA damage response (DDR) that regulates both DNA repair genes and the apoptotic elimination of cells with damaged DNA [21,22]. P53 also has other functions, and it has been questioned whether the DDR is, in fact, responsible for its tumor suppressor function [23,24]. One other function is cell competition, in which damaged cells can be removed only when they are less fit than their neighbors [25–32]. Cell competition removes cells with damage that would be tolerated by cell-autonomous mechanisms [33]. In *Drosophila*, cell competition eliminates cells independently of p53 [34], but in mammals, p53 activities too low to cause apoptosis by cell-autonomous mechanisms can trigger competitive cell removal when compared with less affected surrounding cells [35–40].

In *Drosophila*, the *Xrp1* gene is a potential link between the DNA damage response and cell competition. Xrp1 was first described as a transcriptional target of p53, encodes a *Drosophila* bZip domain protein, and is transcriptionally induced by p53 when *Drosophila* embryos are irradiated [1,2]. Flies lacking wild-type Xrp1 exhibit increased sensitivity to irradiation. Specifically, they show increased loss of heterozygosity in an assay that quantifies patches of cells expressing the recessive *multiple wing hair* (*mwh)* phenotype in otherwise *mwh*^*+/-*^ individuals, indicative of somatic loss of the *mwh*^*+*^ allele [1]. This loss of heterozygosity following irradiation is consistent with a role for Xrp1 in the DDR, downstream of p53.

Xrp1 was later identified as a central regulator of the cell competition of ‘Minute’ (Ribosome protein gene heterozygous mutant) cells in imaginal discs [3,8,9]. Ribosomal protein (Rp) mutant cells provided the first example of cell competition described in *Drosophila* [41]. Most Rp genes are essential, so *Rp* mutations are generally homozygously lethal, whereas *Rp*^*+/-*^ heterozygotes survive with a developmental delay, thin bristles, and some other traits [42,43]. In contrast to whole *Rp*^*+/-*^ animals, individual *Rp*^*+/-*^ cells or clones are eliminated from many tissues when wild-type cells are also present, suffering an enhanced rate of apoptosis [41,44–47]. The Xrp1 protein is expressed at only low levels in wild-type imaginal discs, but Xrp1 protein is robustly induced in *Rp*^*+/-*^ cells, where it is required for transcriptional changes in *Rp*^*+/-*^ wing discs and for many aspects of the *Rp*^*+/-*^ phenotype, including both developmental delay and cell competition [3,8,48]. Xrp1 binds to DNA as a heterodimer with another protein, Irbp18. Unlike Xrp1, Irbp18 is abundantly expressed in wild-type imaginal disc cells [49–51]. The Xrp1/Irbp18 heterodimer activates transcription by binding to a sequence motif identified from both in vitro studies and ChIP-Seq *in vivo* [3,5,52]. Like Xrp1, Irbp18 is also required for cell competition, suggesting that transcriptional targets of the Xrp1/Irbp18 heterodimer are involved [49].

Xrp1 induction in *Rp*^*+/-*^ cells does not require p53 [8]. In *Rp*^*+/-*^ cells, Xrp1 expression is instead induced by a particular Rp, RpS12, which is necessary for cell competition [8,53,54]. Mutations in other *Rp* genes, as well as diminished rRNA synthesis, behave as though they increase RpS12 function [5,53,55]. RpS12 activates Xrp1 expression through alternative splicing of the Xrp1 mRNA to produce an active short isoform [56,57]. Other cellular stresses can also activate Xrp1 expression, at least some independently of both p53 and RpS12, in some cases using other Xrp1 isoforms [5,6,11,33,58–62].

In *Drosophila*, cell competition is part of the response to aneuploidy [13,63–65]. Because the genes encoding 80 Rp are distributed around the chromosomes, loss of chromosome segments constituting more than 1.2% of the genome (on average) typically leads to haploinsufficiency for one *Rp* gene or another by removing one copy of that locus. This leads to the elimination of cells bearing many segmental monosomies by the same cell competition pathway that affects *Rp*^*+/-*^ point mutant cells [13]. Accordingly, *Drosophila* lacking cell competition can accumulate cells with certain kinds of genetic damage, not because they experience any defect in DNA repair, but because some abnormal genotypes that would ordinarily be eliminated by cell competition survive when cell competition is inhibited. Adult flies with a mutation in RpS12 that prevents cell competition exhibit an increased frequency of sporadic cells exhibiting the typical *Rp*^*+/-*^mutant thin bristle phenotype, whether occurring spontaneously or after irradiation. Many of these cells likely harbor chromosome deletions that affect multiple genes in addition to one of the *Rp* loci [13]. Mutations in p53 do not increase the frequency of these putatively aneuploid cells, suggesting that even when DNA repair is impaired, cell competition is sufficient to recognize and eliminate them [13,64].

Because Xrp1 has been implicated in two processes that impact genome integrity, we sought here to define the respective roles more precisely. Loss of heterozygosity after irradiation of Xrp1 mutants could either arise through defects in p53-dependent DNA repair or apoptosis, or due to failure to outcompete cells bearing irradiation-induced segmental aneuploidies. We now report that Xrp1 is indeed important for the DDR, mediating the expression of most of the p53-target genes and contributing to p53-induced apoptosis and the repair or removal of DNA damage. If cells with unrepaired DNA and damaged genomes accumulate after DNA repair, many are subsequently removed by p53-independent, Xrp1-dependent cell competition. Thus, Xrp1 plays at least two roles in genome integrity.

These findings may provide insight into mammalian cancer development. P53 is one of the most important human tumor suppressors, mutated in more than 50% of human cancers [66]. Although p53 ensures genomic integrity as the key effector of the DDR [21,67], it has been questioned whether this is the basis for tumor suppression. Point mutations that block p53 function in the acute DDR have little effect in blocking tumor growth, and conditional knock-out studies point to a critical window for tumor suppressor function that is subsequent to the resolution of DNA damage [24,68–70]. In mammals, which seem to lack a clear Xrp1 ortholog, p53 is required for cell competition, potentially substituting functionally for the role of Xrp1 in *Drosophila* [23,25,71–73]. Cell competition could provide a second role for mammalian p53 in genome integrity, contributing to tumor suppression, if it were found to eliminate aneuploid cells, analogous to the way that Xrp1 contributes to both DDR and cell competition in *Drosophila*.

## Results

### DNA damage-induced expression of Xrp1

**Fig 1:**
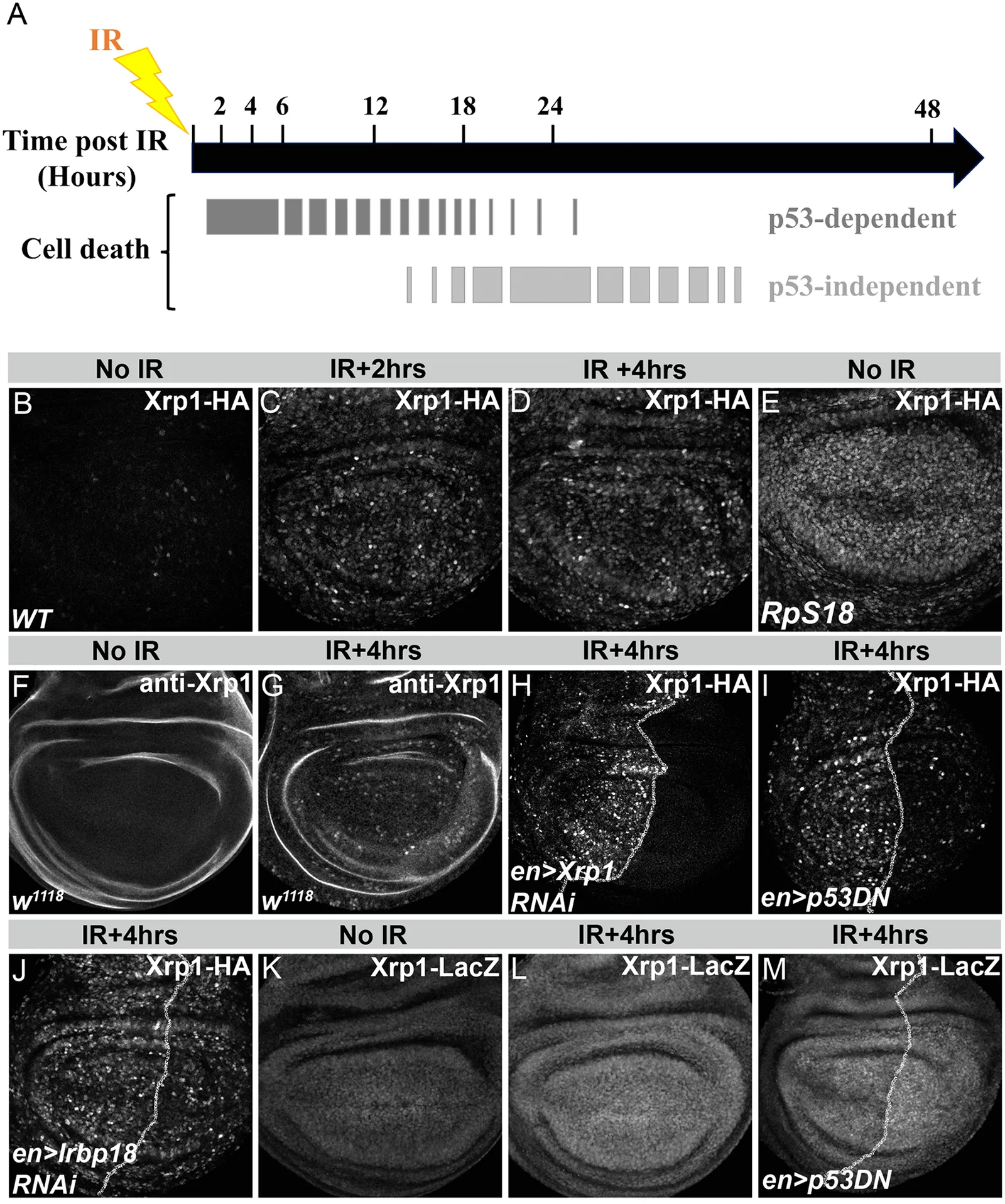
DNA damage induced Xrp1 expression. (A). Schematic of the approximate timeline of p53-dependent and p53-independent apoptotic responses following irradiation, based on previous work [2,13,64,74]. (B). Xrp1 protein levels in the wild type 3RD instar wing discs stained for HA, in 0 Rads (control) (C), 2 hours after 1500 Rads (D), 4 hours after 1500 Rads (E), and in *Rp18*^*+/-*^ mutant (F). Larval wing discs stained with Xrp1 antibody, 0 Rads (control) (G), and 4 hours after 1500 Rads (H) (note: Xrp1 antibody shows higher background than Xrp1-HA). Xrp1-HA expression at 4 hours post-1500 Rads in the posterior compartment, compared relative to the anterior compartment, taken as an internal control, *en-Gal4* driving *UAS-Xrp1* RNAi (I), p53 dominant negative (*UAS-p53*^*H159N*^) (J), and *UAS-Irbp18* RNAi (K). 3rd instar wing discs assayed for *Xrp1*^*02515*^-lacZ enhancer trap expression detected by anti-βgal antibody in 0 Rads (L), 4 hours after 1500 Rads (M), and in the wing disc expressing *UAS-p53*^*H159N*^ under control of *en-Gal4,* 4 hours after 1500 Rads (N). N = 7–8 discs were analyzed for every genotype, and each experiment was repeated at least 2–3 times. Genotypes: (B-D) *Xrp1-HA*, (E) *p{hs:FLP}; FRT42 RpS18 p{ubi:GFP},* (F-G) *w*^*1118*^ (H) *en-Gal4::UAS-RFP, UAS-Xrp1 RNAi* (VDRC 107860)*; Xrp1-HA,* (I) *en-Gal4::UAS-RFP, UAS-p53DN*^*H159N*^ (Bl. 8420)*; Xrp1-HA*, (J) *en-Gal4::UAS-RFP, UAS-Irbp18 RNAi; Xrp1-HA*, (K-L) *P (Meyer et al.) Xrp1*^*02515*^, (M) *en-Gal4::UAS-RFP,UAS-p53DN*^*H159N*^ (Bl. 8420); *P{PZ}Xrp1*^*02515*^.

Studies on irradiated *Drosophila* embryos and larval tissues indicate that DNA Damage-induced apoptosis proceeds in two phases [13,64,74,75]. An initial acute, p53-dependent DDR peaks in the first 4 hours (Fig 1A). This is followed by a p53-independent phase of apoptosis after about 24h thought to include cell competition (Fig 1A). Xrp1 mRNA is upregulated by p53 within 30 minutes of irradiation of *Drosophila* embryos [1,2], perhaps by direct binding of p53 within the Xrp1 locus [76]. We explored Xrp1 mRNA and protein expression to confirm that Xrp1 is also a p53 target in third-instar larval wing discs. We used a fly line in which the Xrp1 protein was expressed from the endogenous locus, containing a C-terminal HA tag [49]. Wild-type wing discs exhibited only a few Xrp1-HA-positive cells, possibly reflecting spontaneous DNA damage or other cellular stress (Fig 1B). Exposure to ionizing radiation (IR: 1500 Rads of γ-rays) induced strongly Xrp1-HA positive cells by 2 hours after IR, which further increased by 4 hours after IR (Fig 1C-1D). The DNA damage-induced Xrp1-HA expression was non-uniform, with some cells showing higher expression levels than others. This heterogeneity was different from the more uniform Xrp1-HA expression seen in *Rp*^*+/-*^ mutant wing discs (Fig 1E). A similar salt-and-pepper pattern of Xrp1 was observed with the Xrp1 antibody (Fig 1F, 1G). The specificity of Xrp1-HA expression was further confirmed by its reduction after RNAi for Xrp1 (Fig 1H).

We tested the requirement for p53 using a dominant-negative form encoded by the *UAS-p53*^*H159N*^ transgene [77,78]. We drove expression in posterior wing compartments so that Xrp1-HA expression levels could be compared to the anterior compartments of the same wing discs as a control. Dominant-negative p53 significantly reduced IR-induced Xrp1-HA (Fig 1I). Xrp1 in wing imaginal disc was also p53-dependent at the transcript level, with a ~ 3x reduction in mRNA level seen after qRT-PCR from irradiated *p53*^*-/-*^ wing discs (S1A Fig).

Interestingly, IR-induced Xrp1-HA protein expression was only slightly affected by Irbp18 knockdown (Fig 1J). This was different from Xrp1 expression in *Rp*^*+/-*^ mutants, where Xrp1 autoregulation is important to regulate the level of Xrp1 expression [3,49]. Consistent with transcriptional autoregulation not contributing to IR-induced *Xrp1* transcription, 50% of the normal *Xrp1* mRNA level remained in the *Xrp1*^*m2-73*^*/Df* mutant background (S1A Fig). Thus, the *Xrp1*^*m2-73*^ point mutant allele could still be transcribed in response to irradiation, even without a functional Xrp1 product.

An Xrp1-LacZ enhancer trap has also been used as a reporter previously. It is upregulated in *Rp*^*+/-*^ mutant wing discs [3,8]. Like Xrp1 protein and mRNA, Xrp1-LacZ was moderately elevated after irradiation (Fig 1K,1L). Different from Xrp1-HA, Xrp1-LacZ expression depended on Xrp1 and Irbp18 function, both after irradiation and in the unirradiated control (S1B-S1E Fig). Unexpectedly, reducing p53 function slightly elevated the expression of Xrp1-LacZ after irradiation (Fig 1M). We can’t completely explain these differences, although they suggest that Xrp1-LacZ might report auto-regulatory Xrp1 transcription but not p53-dependent transcription. We also observed differences between Xrp1-LacZ and endogenous Xrp1 expression in studies of a distinct cell competition mechanism [6].

### Regulation of p53-dependent DDR target genes by Xrp1

**Fig 2:**
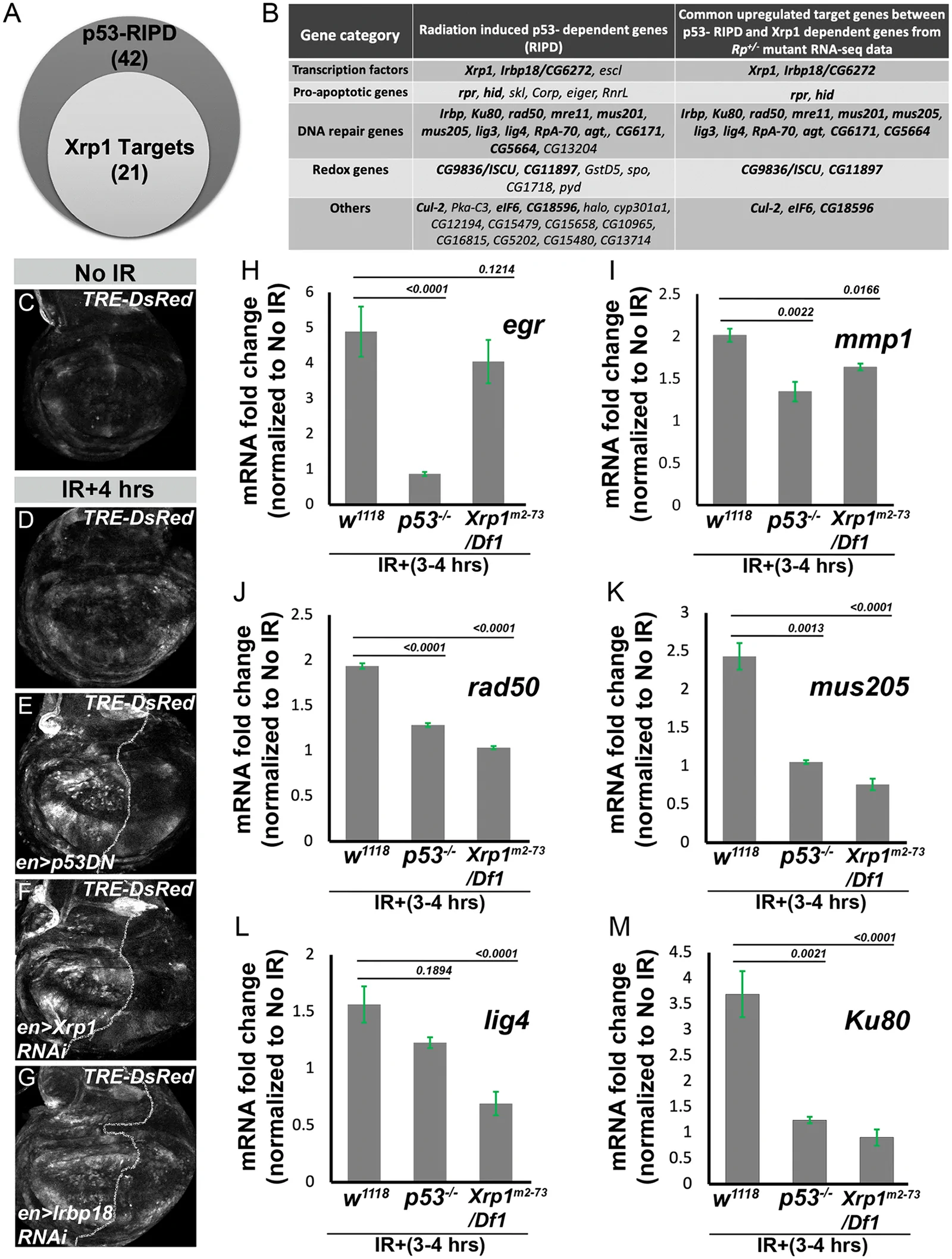
Xrp1 serves as the effector of p53 in DNA damage response. Venn diagram showing the overlap of p53 [1,2] and Xrp1 [3,8] target genes (A). The table shows the genes organized according to GO categories from radiation-induced p53-dependent (RIPD) and genes common between Xrp1- and p53-dependent transcription programs (highlighted in bold) (B). Expression of *TRE-dsRed* in 3RD instar wing discs measured by staining for anti-βgal antibody, in the wild type (no IR) (C), 4 hours after 2000 Rads in wild type disc (D), *UAS-p53*^*H159N*^ (E), *UAS-Xrp1 RNAi* (F), and *UAS-Irbp18* RNAi (G). qRT-PCR analysis for mRNA expression levels from 3RD instar wing discs of *w*^*1118*^, *p53*^*[5A-1-4]*^ and *Xrp1*^*m2-73*^*/Df1* collected 3–4 hours after irradiation (1500 RADs) and normalized to untreated (0 Rads) sample, for the following genes *egr* (H), MMP1 (I), *rad50* (J), *mus205* (K), *lig4* (L) and *Ku80* (M). N = 7–8 discs were analyzed for every genotype, and each experiment was repeated at least 2–3 times. Data are presented as mean ± SEM, two-way ANOVA with Tukey’s multiple comparisons test was used. P < 0.05 was considered statistically significant. Genotype: ((C and D) *TRE-dsRed,* (E) *TRE-dsRed::en-Gal4, UAS-p53*^*H159N*^ (Bl. 8420), (F) *TRE-dsRed::en-Gal4, UAS-Xrp1 RNAi* (VDRC 107860), (G) *TRE-dsRed::en-Gal4, UAS-Irbp18 RNAi*, (H-M) *w*^*1118*^, *yw; p53*^*[5A-1-4]*^*,* and *Xrp1*^*m2-73*^*/Df1*.

In mammals, p53 regulates a complex transcriptional program of hundreds or even thousands of genes [21]. Depending on the study, however, only 122–346 p53-dependent genes have a p53 consensus motif in the promoter or upstream region [79,80]. It is thought that p53 directly controls core transcription programs that, in turn, regulate further secondary, indirect targets [81]. Xrp1 is a candidate for bringing secondary target genes under the indirect control of *Drosophila* p53. Consistent with this, when we compared 42 rapidly induced p53-target genes (RIPD) identified in the DDR [1,2], with Xrp1-dependent genes expressed in *Rp*^*+/-*^ wing imaginal discs or discs overexpressing Xrp1 [3,8], we found 21 of 42 overlapped (Fig 2A). These genes were associated with Gene Ontology (GO) terms: transcription factors, cell death, DNA repair, and redox response (Fig 2B).

We tested the contribution of Xrp1 to p53-dependent gene expression using reporter lines and qRT-PCR. The Jun N-terminal Kinase (JNK) pathway is activated in the DDR [82]. JNK activity is Xrp1-dependent in *Rp*^*+/-*^ wing discs [8,53]. We found the JNK reporter *TRE-dsRed* was induced by IR in a p53-dependent manner (Fig 2C-2E), and this was Xrp1/Irbp18-dependent (Fig 2F & 2G). As expected, the JnK target gene MMP1 was also upregulated in a p53- and Xrp1-dependent manner (Fig 2I). The TNF-alpha ligand *eiger (egr)* has been proposed to activate JNK signaling in the DDR [83]. Surprisingly, however, *egr* transcription was p53-dependent but independent of Xrp1, which questions how much JNK signaling in the DDR depends on *egr* (Fig 2H).

All but one of the p53-dependent DNA repair genes induced in DDR were also potential Xrp1 targets (Fig 2B). We measured wing disc mRNA levels of representative genes from the major DNA repair pathways. These were the homologous recombination pathway gene *rad50*, the translesion polymerase DNA polymerase zeta subunit 1, *mus205*, and the Non-Homologous End Joining (NHEJ) pathway genes DNA ligase 4 (*lig4*) and *Ku80*. We found that IR-dependent expression of all these genes depended on Xrp1 at least as strongly as upon p53 (Fig 2J-2M).

**Fig 3:**
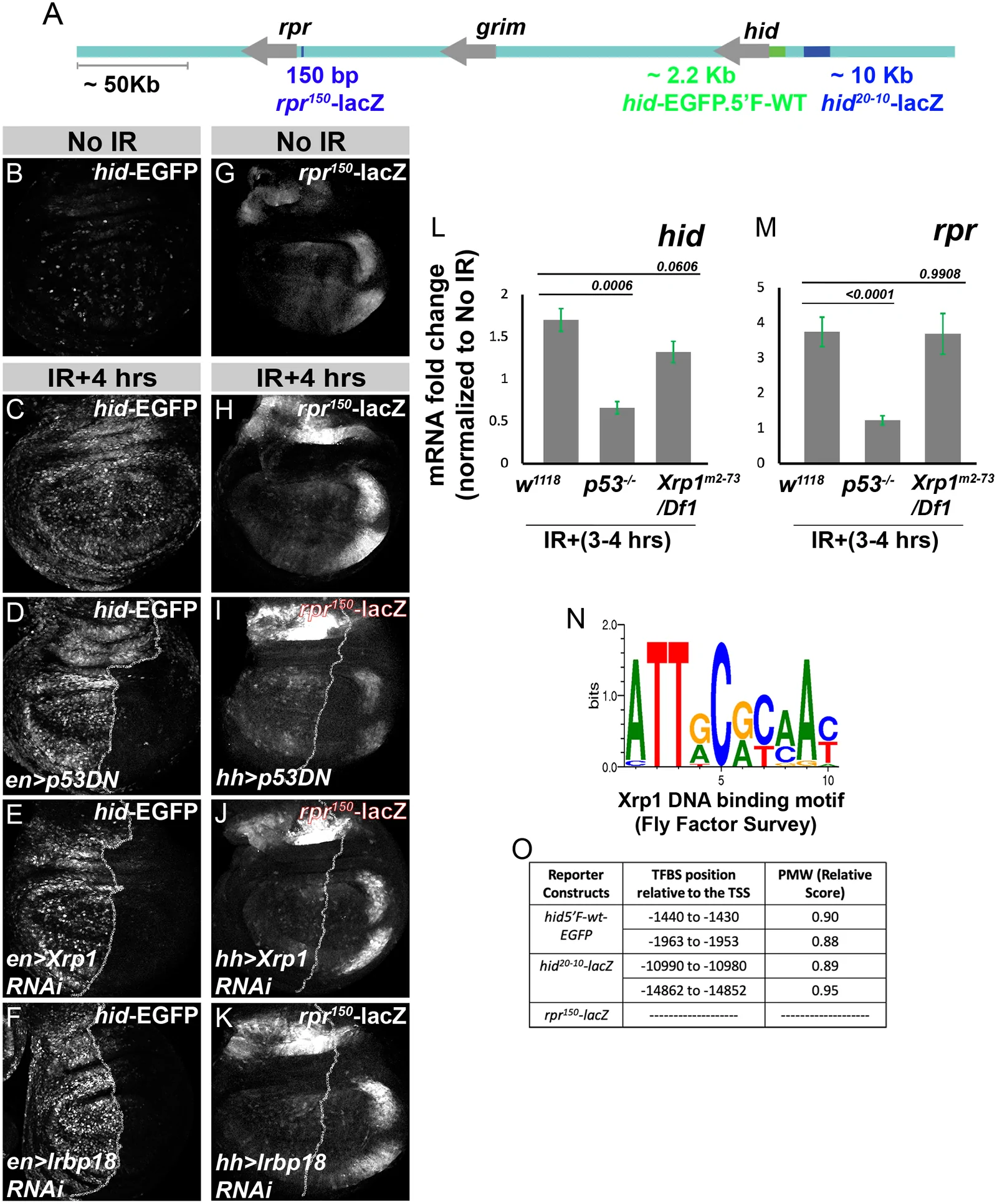
Xrp1 controls the expression of the regulatory sequences of the pro-apoptotic gene *hid.* Schematic of H99 locus with approximate positions of *rpr, hid,* and *grm* and the reporter lines used for the analysis (A). Expression of *hid5’F-wt-EGFP* measured by staining for anti-GFP antibody, in the wild type (no IR) (B) and 4 hours after 2000 Rads in the wild type wing disc (C), *UAS-p53*^*H159N*^ (D), *UAS-Xrp1 RNAi* (E), and *UAS-Irbp18* RNAi (F). *rpr*^*150*^*-lacZ* expression measured by anti-βgal staining, in the wild type (no IR) (G) and 4 hours after 2000 Rads in the wild type wing disc (H). *UAS-p53*^*H159N*^ (I), *UAS-Xrp1* RNAi(J), and *UAS-Irbp18* RNAi (K). qRT-PCR analysis for mRNA expression levels from 3RD instar wing discs of *w*^*1118*^, *p53*^*[5A-1-4]*^, and *Xrp1*^*m2-73*^*/Df1* collected 3–4 hours after irradiation (1500 RADs) and normalized to untreated (0 Rads) sample, for *hid* (L), and *rpr* (M). Putative DNA binding motif of Xrp1/Irbp18 heterodimer, adapted from Fly Factor Survey (N). Position of Xrp1/Irbp18 DNA motif in the upstream regulatory region of *hid* relative to the TSS (O). N = 5–8 discs were analyzed for every genotype. Data are presented as mean ± SEM, two-way ANOVA with Tukey’s multiple comparisons test was used. P < 0.05 was considered statistically significant. Genotype: (B & C) *hid5’F-wt-EGFP***,** (D) *hid5’F-wt-EGFP; en-Gal4::UAS-RFP, UAS-p53*^*H159N*^ (Bl. 8420)**,** (E) *hid5’F-wt-EGFP; en-Gal4::UAS-RFP, UAS-Xrp1 RNAi* (VDRC 107860)**,** (F) *hid5’F-wt-EGFP; en-Gal4::UAS-RFP, UAS-Irbp18 RNAi***,** (G & H) *rpr*^*150*^-lacZ**,** (I) *rpr*^*150*^*-lacZ, UAS-p53*^*H159N*^ (Bl. 8420)*; hh-Gal4::UAS-GFP***,** (J) *rpr*^*150*^*-lacZ, UAS-Xrp1 RNAi* (VDRC 107860)*; hh-Gal4::UAS-GFP,* (K) *rpr*^*150*^*-lacZ, UAS-Irbp18 RNAi; hh-Gal4::UAS-GFP***,** (L-M) *w*^*1118*^, *yw; p53*^*[5A-1-4]*^*,* and *Xrp1*^*m2-73*^*/Df1*.

A well-known aspect of DDR is the apoptotic death of severely damaged cells, dependent in *Drosophila* on p53-dependent induction of the pro-apoptotic genes *rpr* and *hid* [2,84]. To explore the role of Xrp1, we examined reporter lines for *rpr* and *hid* that are expressed in response to IR and widely used as p53 reporters [2,85,86]. The *hid5’F-wt-EGFP* reporter contains ~2 kb spanning the *hid* promoter from +16 to −2210 bp relative to the transcription start site [85,86] (Fig 3A). As expected, *hid5’F-wt-EGFP* was highly induced after IR in a p53-dependent manner (Fig 3B-3D). Both *Xrp1* RNAi and *Irbp18* RNAi reduced the *hid5’F-wt-EGFP* expression to the same extent as p53 dominant-negative did, suggesting that Xrp1 is the direct regulator of this reporter (Fig 3E-3F). Another reporter, *hid*^*20-10*^*-lacZ,* includes a distinct 10 kb sequence from the *hid* upstream region. *hid*^*20-10*^*-lacZ* was only slightly induced by IR, and no effect of *Xrp1* and *Irbp18* RNAi could be detected, although the small IR induction was p53-dependent (S2D-S2E and S2F-S2G Figs). Next, we assessed *rpr* expression using the *rpr*^*150*^*-lacZ* reporter, containing a 150 bp sequence including a putative p53 binding site ~4.8kb upstream of the *rpr* gene [87], and which has been used as a p53-reporter in wing discs [88]. In controls, *rpr*^*150*^*-lacZ* reporter exhibits uniform expression in most of the wing disc, on which is superimposed a region of elevated expression bordering the posterior wing pouch and notum region [89,90] (Fig 3L). It is now appreciated that many DDR genes have non-uniform expression patterns in the wing disc [91]. Because IR enhanced all aspects of *rpr*^*150*^*-lacZ* expression (Fig 3G,3H), we measured the average *rpr*^*150*^*-lacZ* expression level in anterior and posterior compartments and computed the ratio of posterior-to-anterior expression level as a readout of genetic perturbations applied to the posterior compartments. The increase in *rpr*^*150*^*-lacZ* following IR was p53-dependent, as expected, but unaffected by RNAi for either Xrp1 or Irbp18 (Fig 3G-3K and S2H-S2I Fig).

To confirm that these studies of *hid* and *rpr* reporters were indeed reflective of the expression of the genes themselves, mRNA levels were measured in wing discs after IR. Consistent with the reporter studies, induction of *hid* mRNA by irradiation was completely dependent on p53 and partially dependent on Xrp1 (Fig 3L). Induction of *rpr* mRNA expression was completely dependent on p53 but was unaffected by the loss of Xrp1 (Fig 3M).

Taken together, these results show that genes identified as p53 targets in the *Drosophila* DDR include many that are potentially induced directly by Xrp1. Only two out of nine p53 target genes examined, *egr* and *rpr*, were induced independently of Xrp1. Analysis of p53 and Xrp1 binding motifs supported this conclusion. Sequences matching the p53-binding consensus have been identified in both the *rpr*^*150*^*-lacZ* reporter and the *hid5’F-wt-EGFP* reporter [87,92]. Consensus binding sites for the Xrp1/Irbp18 heterodimer [3,52] (Fig 3O) are footprinted by Xrp1/Irbp18 in the P-element terminal repeat and are sufficient to confer Xrp1/Irbp18-dependent transcription on luciferase reporters {[5] #4653; [50] #3670}. Using an *in-silico* motif finder (INSECT) [93], we found two Xrp1/Irbp18 binding motifs in the *hid5’F-wt-EGFP* reporter sequence and two in the *hid*^*20-10*^*-lacZ* reporter sequence (Fig 3P), but none in the *rpr*^*150*^*-lacZ* reporter that was expressed independently of Xrp1 and Irbp18 (Fig 3P). We conclude that, while *rpr* could be a direct target of p53 in the DDR, *hid* expression is partially dependent on Xrp1 induction downstream of p53, and the *hid5’F-wt-EGFP* reporter entirely so.

### Xrp1 is sufficient for the expression of many p53-dependent DDR target genes

**Fig 4:**
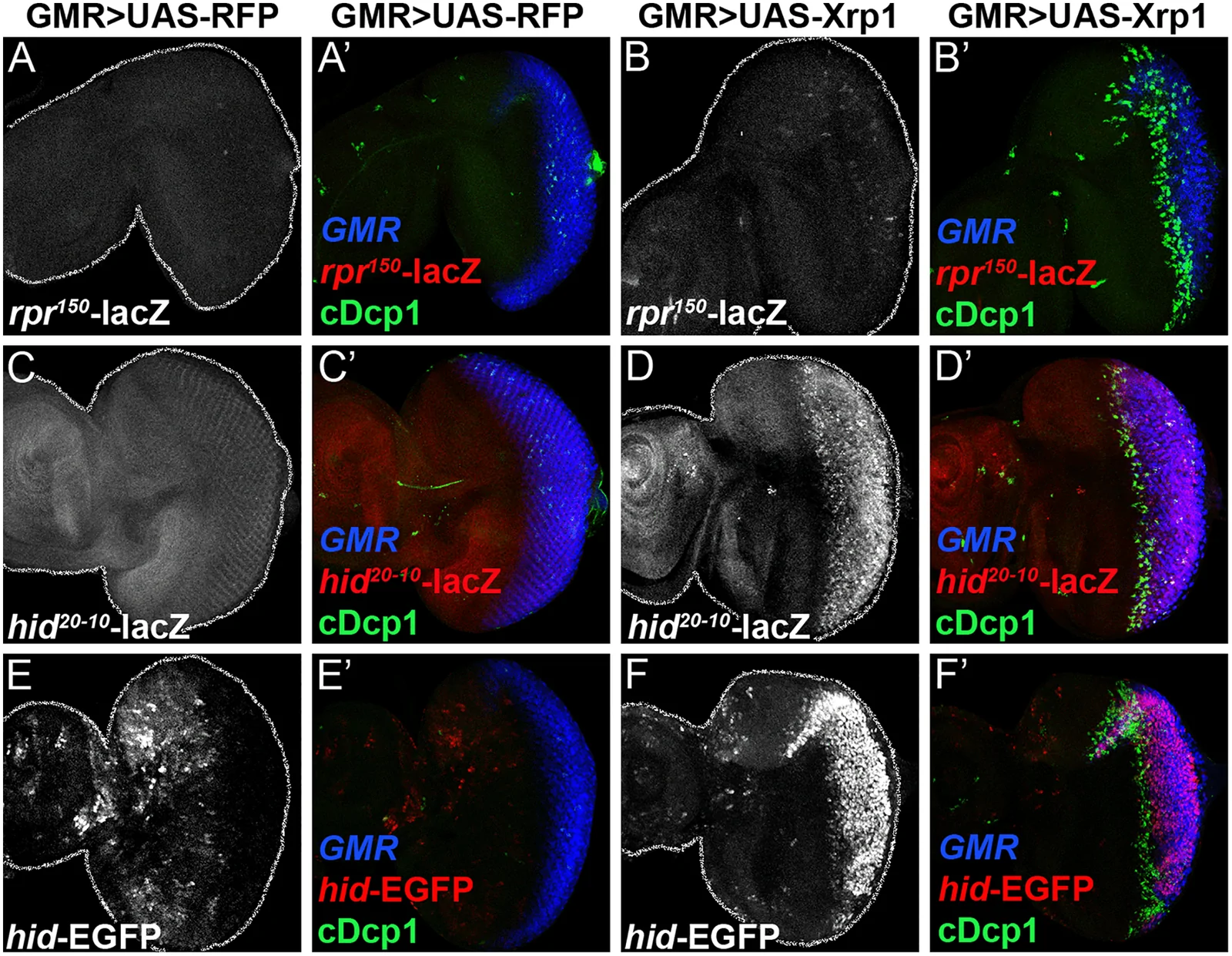
Xrp1 is sufficient for the expression of many p53- dependent DDR target genes. *rpr*^*150*^*-lacZ* reporter expression measured by anti-βgal (red) and cell death by anti-Dcp-1 (green) in 3RD instar eye imaginal disc expressing UAS-RFP (A) and UAS-Xrp1 long isoform (B), under the control of *GMR-Gal4* (blue). *hid*^*20-10*^*-lacZ* reporter expression measured by anti-βgal (red) and cell death by anti-Dcp-1 (green) in 3RD instar eye imaginal disc expressing UAS-RFP (C) and UAS-Xrp1 long isoform (D) under the control of *GMR-Gal4* (blue). *hid5’F-wt-EGFP* reporter expression measured by anti-GFP (red) and cell death by anti-Dcp-1 (green) in 3RD instar eye imaginal disc expressing UAS-RFP (E) and UAS-Xrp1 long isoform (F) under the control of *GMR-Gal4* (blue). N = 7–8 discs were analyzed for every genotype; each experiment was repeated at least 2 times. All the control and experimental genotypes shown in this figure were kept at 18°C to lessen the effect of Xrp1 overexpression. Genotype: (A-A’) *GMR-Gal4::UAS-RFP, rpr*^*150*^*-lacZ,* (B-B’) *GMR-Gal4::UAS-RFP::UAS-Xrp1, rpr*^*150*^*-lacZ,* (C-C’) *GMR-Gal4::UAS-RFP; hid*^*20-10*^*-lacZ,* (D-D’) *GMR-Gal4::UAS-RFP::UAS-Xrp1; hid*^*20-10*^*-lacZ,* (E-E’) *hid5’F-wt-EGFP; GMR-Gal4::UAS-RFP*, (F-F’) *hid5’F-wt-EGFP; GMR-Gal4::UAS-RFP, UAS-Xrp1*.

It was possible that the only Xrp1 requirement in the DDR might be to activate p53 to create a positive feedback loop. We used the *rpr*^*150*^*-lacZ* reporter, which is activated by p53 but not Xrp1, to test how p53 activity was affected by Xrp1 overexpression. Xrp1 overexpression in the wing pouch resulted in a huge amount of cell death, as expected (S3A-S3B Figs), but no discernible increase in *rpr*^*150*^*-lacZ* levels (S3C-S3D Figs). Similar results were obtained by expressing Xrp1 in the eye discs (Fig 4A-4B). Thus, Xrp1 did not cause p53 activation and is likely to act downstream of p53.

Next, we tested if Xrp1 was sufficient for the expression of target genes. Xrp1 overexpression in the eye disc led to a robust upregulation of *hid*^*20-10*^*-lacZ* (Fig 4C and 4D) and *hid5’F-wt-EGFP* (Fig 4E and 4F). Xrp1 also regulated JNK after DNA damage, and Xrp1 overexpression was sufficient to activate the JNK reporter *TRE-dsRED* (S3E-S3F Figs). These results show that Xrp1 functions downstream of p53 and is sufficient for expressing a subset of p53-dependent DDR genes.

### Xrp1 contributes to DNA damage-induced cell death

**Fig 5:**
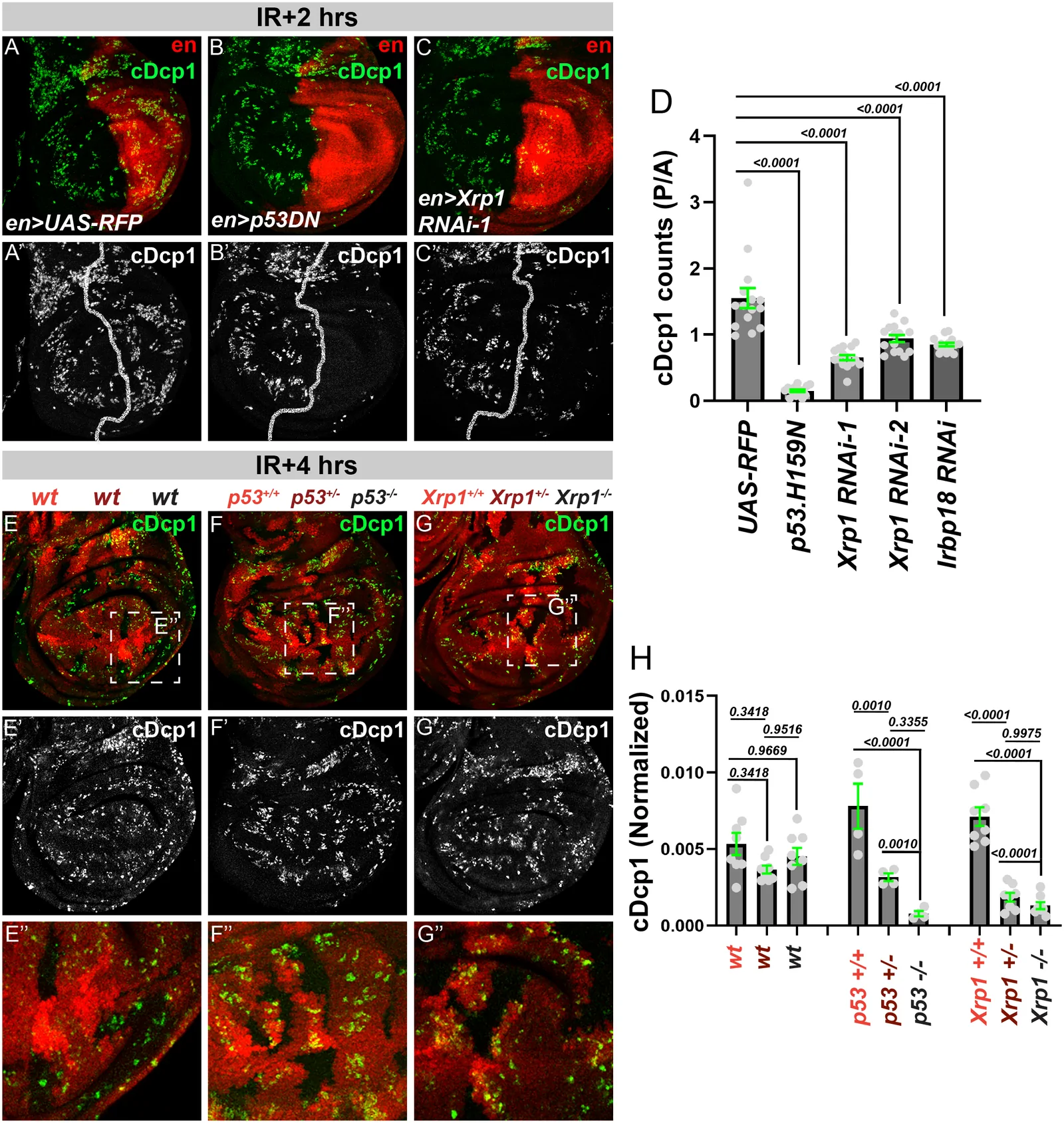
Xrp1 is partly required for DNA damage-induced cell death. *Drosophila* 3RD instar wing imaginal discs dissected at 2 hours after 1500 Rads, cell death is measured by Dcp-1 (green and grayscale) in different genetic perturbations using en-Gal4 (posterior compartment (red)), UAS-RFP (A), *UAS-p53*^*H159N*^ (B), and *UAS-Xrp1* RNAi (C). Dcp-1 positive cells were quantified and plotted as the ratio of Dcp-1 positive cells in posterior (P) to anterior (A) compartments (D). Wing discs were dissected 4 hours after 1500 RADs and stained for Dcp-1 positive cells in *FRT82* (E), *FRT82p53*^*[5A-1-4]*^ (F), and *FRT82Xrp1*^*m2-73*^ (G). (H) Dcp-1 positive cells were quantified in the clone (RFP-/-), heterozygous tissue (RFP + /-) and in the sister clone (RFP + /+), 4-hour after IR. (E”-G”) display the magnified image of boxed area. Data are presented as mean ± SEM, two-tailed Welch’s t-test was used, and one-way ANOVA with Tukey’s multiple comparisons test was used. P < 0.05 was considered statistically significant. Genotype: (A) *en-Gal4::UAS-RFP,* (B) *en-Gal4::UAS-RFP, UAS-p53DN*^*H159N*^ (Bl. 8420), (C) *en-Gal4::UAS-RFP, UAS-Xrp1 RNAi* (Bl. 34521), (E) *p{hs:FLP}; FRT82-RFP/FRT82* (30 min hs, 48 hr A.E.L)*,* (F) *p{hs:FLP}; FRT82-RFP/FRT82-p53*^*[5A-1-4]*^ (30 min hs, 48 hr A.E.L), (G) *p{hs:FLP}; FRT82-RFP/FRT82-Xrp1*^*m2-73*^ (30 min hs, 48 hr A.E.L).

To see whether the regulation of cell death genes by Xrp1 was functionally significant, the *p53*-dependent apoptosis that occurs in response to irradiation was examined. Cell death begins within 30 minutes after IR treatment and is entirely dependent on p53 for at least 4–5 hours [16,64]. Accordingly, p53 dominant-negative expression in posterior compartments substantially reduced the IR-induced cell death, leaving only a few cells positive for the cleaved Dcp-1 caspase (Fig 5A, 5B and 5D). Xrp1 RNAi (two independent lines) and Irbp18 RNAi both reduced cell death in the posterior compartment (Fig 5C-5D), although to a lesser extent than p53 dominant-negative did.

We also examined the cell autonomy of p53 and Xrp1 function using clones of mutant cells. Control clones did not affect IR-induced cell death (Fig 5E and 5H). Cell death was reduced in *p53*^*+/-*^ heterozygous cells and almost absent from *p53*^*-/-*^ null clones after irradiation (Fig 5F and 5H). Cell death was also significantly reduced in *Xrp1*^*+/-*^ (heterozygous) and even more so in *Xrp1*^*-/-*^ (null) tissues as compared to *Xrp1*^*+/+*^ (wild type twin spot) (Fig 5G and 5H) (effects of Xrp1 mutation on *Rp* mutant cells are also dominant through haploinsufficiency) [8]. The *Xrp1*^-/-^ mutant genotype reduced cell death to a greater extent than Xrp1 RNAi, although still not as much as *p53*^*-/-*^ loss of function. Taken together, these results show that Xrp1 contributes to the p53-mediated cell death in response to irradiation (Fig 3R).

We also tested the contribution of Xrp1 to the effects of p53 overexpression. p53 overexpression in wing discs resulted in massive cell death that was partially but significantly reduced by Xrp1 knockdown or mutant genotypes (S4A-E and G Figs). In accordance with this partial dependence on Xrp1, p53 overexpression led to rather little Xrp1 protein expression (S4F Fig). Over-expression of p53 in the developing eye causes a rough eye phenotype in adults (S4H Fig) [77,78]. Xrp1 knockdown very slightly reduced the rough eye phenotype, unlike dominant-negative p53, which rescued it completely (S4I and S4J Figs).

### RpS12-induced Xrp1 limits genomic instability after the DDR

The DDR is followed by a phase of p53-independent cell death, peaking around 24h after irradiation [75]. This has been attributed to the competitive loss of aneuploid cells mediated by altered Rp gene dose [64]. Consistently, irradiation leads to phenotypically Minute adult bristles, most of which are likely segmental monosomies [64]. Our previous studies supported this hypothesis [13]. We found that more Minute bristles accumulated when cell competition was blocked by mutating *rpS12* [13]. We found that 66% of this delayed, p53-independent cell death that follows irradiation depended on *rpS12*; by contrast, *rpS12* is not required for p53-dependent apoptosis in the DDR that peaks around 4h after IR, also confirmed here (S5A-S5C Figs) [13]. Consistent with the role of rpS12 being to initiate Xrp1-dependent cell competition of cells with a deficient dose of one or more *Rp* genes, Xrp1 was induced independently of p53 but most (58%) depended on *rpS12* [13]. These findings were as expected if cell competition on the basis of altered *Rp* gene dose activates Xrp1 through RpS12, as would be expected of cells that acquire significant monosomies after DNA damage [13].

**Fig 6:**
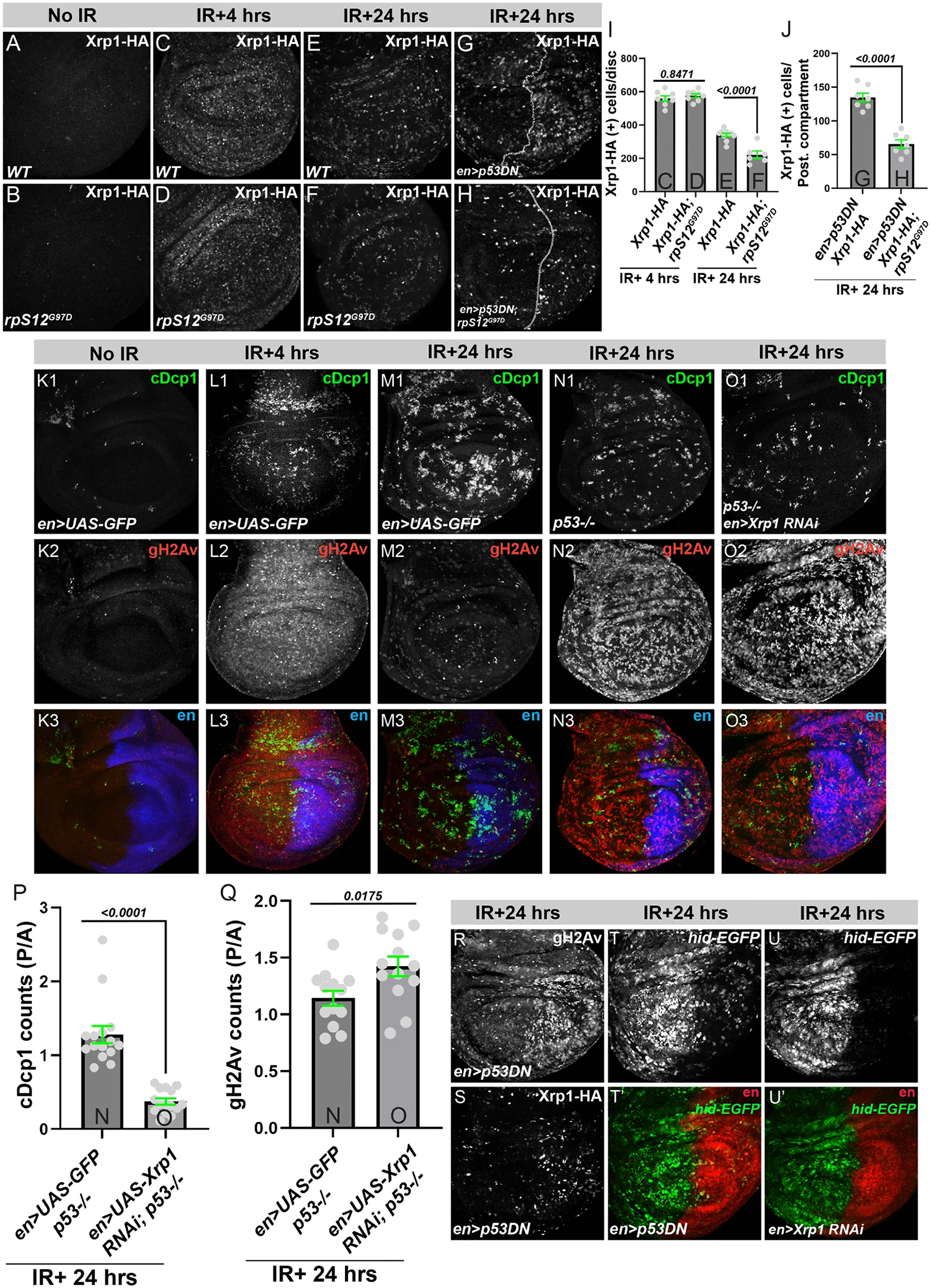
Xrp1 limits genomic instabilities by removing aneuploid cells in a p53-independent manner. (A-H) Xrp1-HA expression in control disc no IR (A), Xrp1-HA expression in *rpS12*^*G97D/G97D*^ mutant background no IR (B), Xrp1-HA expression 4 hours after 1500 rads in control disc (C), Xrp1-HA expression 4 hours after 1500 rads in *rpS12*^*G97D/G97D*^ mutant background (D), Xrp1-HA expression 24 hours after 1500 rads in control disc (E), Xrp1-HA expression 24 hours after 1500 rads in *rpS12*^*G97D/G97D*^ mutant background (F). Xrp1-HA expression 24 hours after 1500 rads in en > *UAS-p53*^*H159N*^ (G). Xrp1-HA expression 24 hours after 1500 rads in en > *UAS-p53*^*H159N*^ plus *rpS12*^*G97D/G97D*^ mutant background (H). (I) Quantification of Xrp1-HA positive cells 4 and 24 hours after IR in control (C, E) and *rpS12*^*G97D/G97D*^ mutant background (D, F). (J) Quantification of Xrp1-HA positive cells in posterior (Post.) compartments in *UAS-p53*^*H159N*^ and *UAS-p53*^*H159N*^ in *rpS12*^*G97D/G97D*^ mutant background*.* (K-L) 3RD instar wing disc from larvae stained for cell death (Dcp-1, green), DNA damage signaling (γH2Av, red), control (*en-Gal4, UAS-GFP*) (K), and 4 hours after 1500 Rads of γ-rays treatment (L), (M-O) 3RD instar wing disc from larvae 24 hours after 1500 Rads of γ-rays treatment, stained for cell death (Dcp-1, green), DNA damage signaling (γH2Av, red) in control (*en-Gal4, UAS-GFP*) (M), *p53*^*-/-*^ mutant (N), and Xrp1 k/d in *p53*^*-/-*^ mutant background (O). (P-Q) Quantification of cDcp-1-positive (P) and γH2Av (Q) cells in anterior versus posterior compartments in *p53*^*-/-*^ mutant and Xrp1 k/d in *p53*^*-/-*^ mutant background. (R) γH2Av and (S) Xrp1-HA levels 24 hours after irradiation in the posterior compartment of the wing disc expressing *p53*^*H159N*^ under the control of engrailed Gal4*. hid5’F-wt-EGFP* expression in *UAS-p53*^*H159N*^ (T) and Xrp1 RNAi K/D (U) 24 hours after irradiation. N = 8 for (C & D), N = 10 for (E), N = 6 for (F), N = 8 for (G & H), and N = 15 for (N-O). (E). Data are plotted as mean ± SEM. One-way ANOVA with Tukey’s multiple comparisons test was used for (C-F), and a two-tailed Student’s t-test for independent samples for (G-H and N-O) was performed to assess statistical significance. Genotype: (A, C, E and G) *Xrp1-HA*, (B, D, F, H) *Xrp1-HA-rpS12*^*G97D/G97D*^*,* (I) *en-Gal4::UAS-RFP; UAS-p53DN*^*H159N*^ (Bl. 8420)*; Xrp1-HA,* (J) *en-Gal4::UAS-RFP; UAS-p53DN*^*H159N*^ (Bl. 8420)*; Xrp1-HA-rpS12*^*G97D/G97D*^*,* (K-L) *en-Gal4::UAS-GFP*, (M) *en-Gal4::UAS-GFP; FRT82-RFP/FRT82-p53*^*[5A-1-4]*^, (N) *en-Gal4::UAS-GFP; FRT82-RFP/FRT82-p53*^*[5A-1-4]*^, (O) *en-Gal4::UAS-GFP, UAS-Xrp1 RNAi* (VDRC 107860)*; FRT82-RFP/FRT82-p53*^*[5A-1-4]*^*,* (R) *en-Gal4::UAS-RFP; UAS-p53DN*^*H159N*^ (Bl. 8420) (S) *en-Gal4::UAS-RFP; UAS-p53DN*^*H159N*^ (Bl. 8420)*; Xrp1-HA*, (T) *hid5’F-wt-EGFP; en-Gal4::UAS-RFP, UAS-p53*^*H159N*^ (Bl. 8420)**,** and (U) *hid5’F-wt-EGFP; en-Gal4::UAS-RFP, UAS-Xrp1 RNAi* (VDRC 107860).

Here we have extended these studies, focusing on the functional contributions of Xrp1 and p53. As reported previously using an antibody against Xrp1 [13], we found that p53-independent cell death 24h post-IR was associated with Xrp1-HA expression. Just as found with Xrp1 antibody, more than half of the p53-independent Xrp1-HA expression depended on RpS12 (Fig 6E-6F & 6J). Xrp1-HA expression was also reduced in the *rpS12*^*G97D*^ mutant when p53 was wild-type, although to a lesser degree, presumably because p53-dependent Xrp1 expression does not depend on *RpS12* (Fig 6G-6H & 6I). We now find that 65% of p53-independent apoptosis 24h post-IR was Xrp1-dependent (Fig 6N- 6P), comparable to the 66% that was previously found to be RpS12- dependent [13]. Xrp1 also contributed to the total amount of apoptosis that occurred when p53 was not mutated (S5D-S5E Figs). These findings are consistent with the idea that delayed, p53-independent apoptosis 24h after irradiation includes competitive elimination of many cells containing aneuploidies that reduce Rp gene dose.

Unexpectedly, when p53 Inhibition was limited to posterior compartments so that anterior compartments of the same discs served as internal controls, it was clear that not only was much of the Xrp1 expression 24h post-IR independent of p53, but that it was actually increased by p53 inhibition (Fig 6G). Some of this increase was rpS12-dependent (Fig 6G-6H & 6J), consistent with more cell competition occurring after p53 inhibition. Since cell competition of *Rp*^*+/-*^ cells is not affected by p53 in *Drosophila* [34], this is more likely explained by the presence of more *Rp* haploinsufficient cells when p53 had been inhibited during the DDR.

To investigate how p53 inhibition impacts genome integrity further, we looked at IR-induced DNA damage using antibodies against γH2Av, which marks the double-strand breaks [94,95]. Consistent with previous reports, the acute DNA damage revealed by γH2Av staining 4h after IR was mostly repaired by 24 hours post IR (Fig 6K-6M) [64,74]. This was not true in the absence of p53, however: γH2Av levels remained high, indicating persistent double-strand breaks (Fig M,N). Dual Xrp1 knockdown and p53 inhibition led to an even further increase (Fig 6O,6Q), and there was also a small but reproducible elevation of γH2Av levels when only Xrp1 was depleted (S5D and S5F Figs). These data indicated that p53 was required for the full repair of irradiation-damaged DNA (or for the removal of some cells with incomplete repair). Some cells where this damage persists were then removed or repaired by p53-independent Xrp1 activity, to which cell competition might contribute (Fig 6R-6S). Another indication of a role of Xrp1 independent of p53 came from *hid5’F-wt-EGFP* expression, which was totally dependent on Xrp1 24h after IR, but only partially dependent on p53 (Fig 6T-6U). Because Xrp1 function in the DDR is downstream of p53, this role of Xrp1 in p53 mutants may instead reflect the late, p53-independent function that also removes aneuploid cells. Importantly, aneuploid cells are not expected to label for γH2Av, because segmental aneuploidy arises when DNA breaks are joined inappropriately, not left unrepaired. Instead, persistent γH2Av labeling is a sign of delayed or defective repair processes that might also lead to more aneuploidy. Unfortunately, there is currently no straightforward assay that can identify all the aneuploid cells in imaginal discs; RpS12-dependent Xrp1 induction may be a useful surrogate marker for aneuploidy.

To get a better idea of when cell competition begins, wing discs were examined 16 hours post-IR. We found that most Xrp1-HA expression was independent of p53 by this stage (S5G-S5I Figs). Similar to 24 hours, Xrp1-HA was even higher in the absence of p53. However, similar to 4 hours after IR, Xrp1 knockdown suppressed Xrp1-HA expression at 16 hours post-IR, but Irpb18 did not (S5J, S5K Figs). Cell death occurring 16 hours after irradiation was substantially p53-dependent, although p53-independent cell death was also detected (S6L, S6M and S6O Figs). Cell death is almost completely p53-dependent at the acute DDR stage (Fig 5). Cell death 16 hours following irradiation was reduced by Xrp1 and Irbp18 depletion, but not eliminated (S5L, S5N and S5O Figs). These results suggest that p53-independent Xrp1 expression and function in cell competition are emerging by 16 hours and peaking by 24 hours after irradiation, perhaps indicating the timeline of the appearance of aneuploid cells.

## Discussion

**Fig 7:**
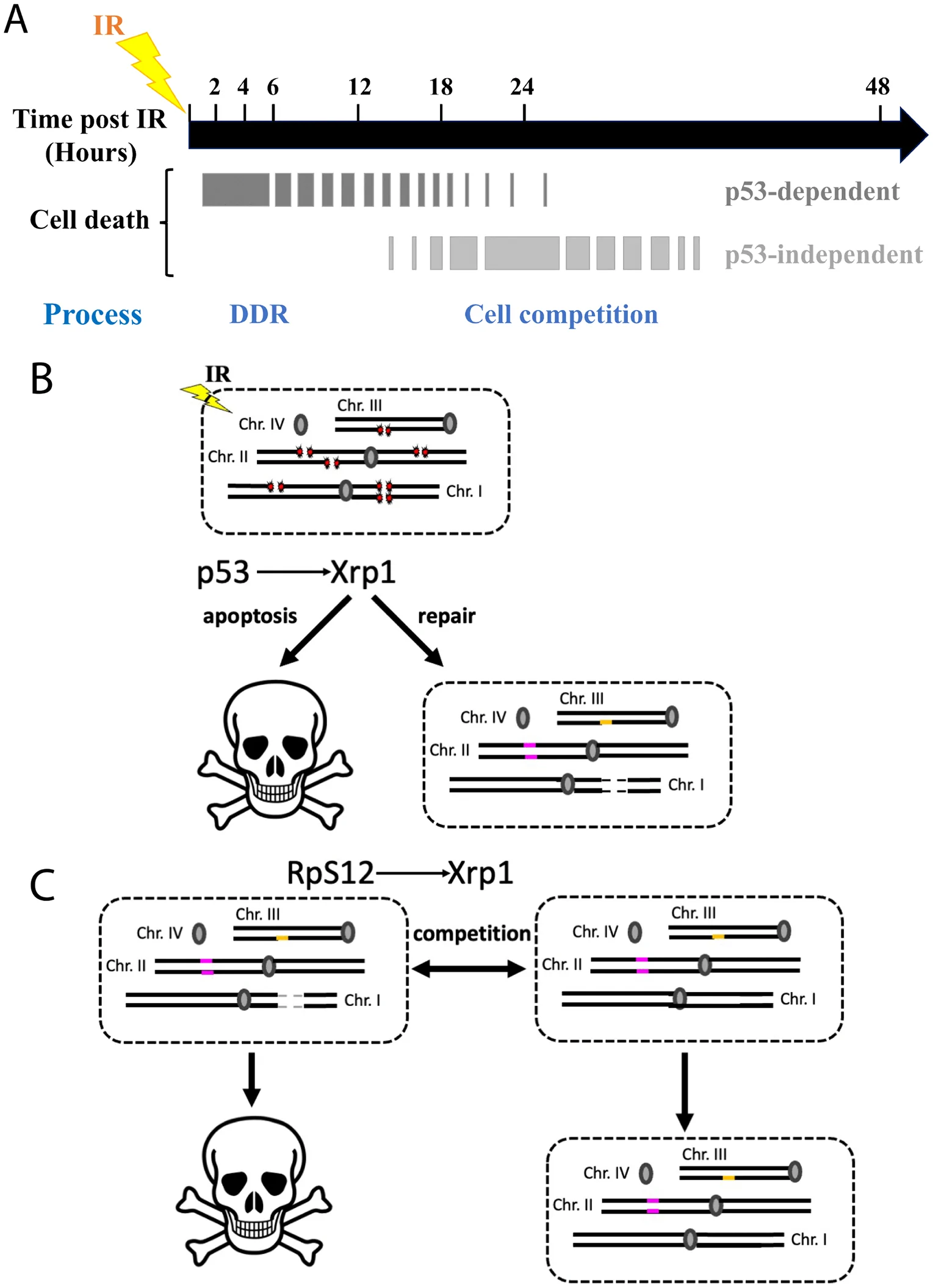
Model. (A) Our work reinforces the conclusion that an acute, p53-dependent apoptotic response to irradiation represents the DDR, while the later, p53-independent phase is associated with elimination of aneuploid cells by cell competition. The p53-independent phase depends more completely on Xrp1, whose expression at this stage is RpS12-dependent. The cell competition response is enhanced when p53 is absent, resulting in prolonged DNA damage and presumably an increase in aneuploidy. (B) The acute, p53-dependent DDR acts in part through Xrp1 as a downstream transcription factor. The DDR results in the repair of DNA damage or the removal of damaged cells by cell death. DNA repair can introduce errors (colored) including chromosome aberrations resulting in loss of genetic material (dashes). (C) When repair of DNA damage results in segmental aneuploidy, Xrp1 activation is triggered via an RpS12-dependent, p53-independent pathway that removes aneuploid cells via cell competition with unaffected cells.

Xrp1 is known to contribute to genome integrity in *Drosophila*. Flies lacking Xrp1 exhibit increased loss-of-heterozygosity following irradiation, and also accumulate thin, Minute-like adult bristles indicative of monosomy for regions including *Rp* genes [1,13]. Here we affirm that Xrp1 contributes to genome maintenance at two levels (Fig 7). One is as a component of the acute DDR, mediating part of the p53 transcriptional response and p53-dependent apoptosis; the other role occurs later and appears to include cell competition to eliminate aneuploid and segmentally aneuploid cells that have acquired altered Rp gene dose as a result of DNA damage and its subsequent repair (Fig 7A). These two roles synergize to maintain somatic genome integrity after damage. They are distinguished in *Drosophila* by their mechanisms of induction. In the DDR, Xrp1 is induced transcriptionally by p53. RpS12 induces Xrp1 during cell competition. In mammals, by contrast, both the DDR and cell competition depend on p53 and are both potential contributors to p53-mediated tumor suppression.

### Xrp1 is an effector of p53 in the DDR

In imaginal discs, Xrp1 is induced by p53 activity in the acute phase of DDR at both RNA and protein levels (Fig 1). Unlike Xrp1 induction in *Rp*^*+/-*^ cells, Xrp1 expression in the DDR occurs independently of autoregulatory transcription, which may be why the Xrp1-LacZ enhancer trap shows less upregulation than the endogenous Xrp1 transcript and protein. Multiple p53-dependent DDR genes, in fact, the majority in our study, were Xrp1-dependent, including the pro-apoptotic gene *hid*. Even though *the hid5’F-wt-EGFP* reporter has been used as a readout of p53 activity [85,96–98], it may be more accurately described as a target of Xrp1. Other Xrp1 targets in the DDR include DNA repair genes and JNK signaling. These roles of Xrp1 were functionally required in the DDR. DNA damage-induced cell death was reduced in the absence of Xrp1, although not eliminated, perhaps because the pro-apoptotic gene *rpr* is a direct target of p53 that does not require Xrp1. Double strand break repair was also diminished when Xrp1 was depleted.

These findings confirmed that, in *Drosophila*, Xrp1 acted downstream to p53 as an intermediate transcription factor and controls many DDR targets. So far, integrating data from gene expression studies, binding motif and ChIP-Seq searches, and global run-on sequencing of nascent mRNA has not yielded a consensus on the core set of direct p53 target genes in mammals [79,99,100]. It is possible that, as in *Drosophila*, mammalian p53 acts as a master transcription factor that regulates a network of gene expression responses both directly and through intermediate transcription factors.

### Xrp1 also controls genome integrity independently of p53 and the DDR

Although Xrp1 protein expression was almost completely p53-dependent during the acute DDR response, later Xrp1 expression was mostly p53-independent (S5 Fig). DNA damage is normally resolved by the time of p53-independent Xrp1 expression [74]. Without p53, however, DNA damage persisted longer than usual, so γH2Av labeling was still evident 24 h after irradiation (Fig 6). Persistent γH2Av staining may reflect ineffective DNA repair, insufficient apoptosis that permits badly damaged cells to survive, or both.

Previous work suggests that p53-independent cell death includes cell competition of cells whose genomes have been altered by irradiation. Because 80 Rp are encoded by genes spread throughout the genome, a loss of 1–2% of the genome will often create haploinsufficiency for one or more Rp genes and trigger elimination by cell competition [13]. Consistent with this mechanism, the delayed, p53-independent Xrp1 expression largely depended on RpS12, a signal of defective ribosome biogenesis that is required to induce Xrp1 in *Rp*^*+/-*^ cells and cells with defective rRNA synthesis [5,8]. Accordingly, the p53-independent apoptosis occurring 24 hours after IR, and which is known to depend on RpS12, also depended on Xrp1 (Fig 6E, 6F, 6I). Xrp1 thus contributes to genome integrity at two levels, as a component of the p53-dependent DDR and later in a p53-independent manner, including RpS12-dependent cell competition that may remove cells where DDR has failed or has altered cell genotypes (Fig 7B,7C). RpS12-dependent cell death may depend on competition from neighboring cells because direct targets of p53, which include *rpr*, are not induced. In addition, since *rpS12* is thought to affect targets besides Xrp1 [53,57], other *rpS12* targets might increase the role of neighboring cells in cell competition.

Xrp1 expression levels were even further elevated during the cell competition phase if p53 had been inhibited (S6 Fig). Because this enhanced Xrp1 expression was p53-independent and at least partly RpS12-dependent, it was likely to include cell competition of aneuploid cells. This suggested that more aneuploid cells arose when p53 was not available to coordinate the DDR. γH2Av labeling also suggested that double-strand breaks persisted without p53. Importantly, γH2Av labeling is not itself a signature of aneuploid cells but reflects delayed and defective DNA repair processes that may correlate with increased aneuploidy. We think that without p53, DNA repair pathways, including DSB repair, single-strand break repair, and nucleotide excision repair, are delayed or less effective, and coupled with faulty cell cycle arrest, result in a higher frequency of genome rearrangements and aneuploidy [101].

### Dual processes maintaining genome integrity following DNA damage

We have focused on two Xrp1-dependent processes contributing to genome integrity after DNA damage. The DDR depends on p53 in both *Drosophila* and mammals. Cell competition removes aneuploid cells in a p53-independent but rpS12-dependent manner in *Drosophila*, but is p53-dependent in mammals (Fig 7).

In mammals, the DDR was initially thought to reflect the tumor-suppressor function of p53. Later studies showed that tumor suppression is separable from the DDR and occurs afterward [24,68–70]. In mammals, where no clear Xrp1 homolog exists, Rp gene haploinsufficiency instead induces p53 through the nucleolar stress pathway [102]. Interestingly, differences in p53 activity levels between cells underlie multiple examples of cell competition in mammals [35,37–39]. Accordingly, we have suggested that in mammals, p53 takes on cell competition aspects of the Xrp1 function in *Drosophila* [71]. Consistent with this, p53 mediates the removal of aneuploid cells in mammals, which Xrp1 does in *Drosophila* [13,103]. If p53 eliminates aneuploid cells in mammals by Rp gene dose-dependent cell competition, as Xrp1 does in *Drosophila*, then dual roles for mammalian p53 could both contribute to tumor suppression, as dual roles for Xrp1 maintain genome integrity in *Drosophila*. Consistent with this, analyses of early cancer development in a mouse pancreatic cancer model indicate that losses of single chromosomes or parts of chromosomes are very early steps in the progression toward transformation [104]. Mammalian cells with such small-scale monosomies only seem to survive in the absence of p53 [105,106]. Although further studies will be required to confirm the suggestion, the observations are consistent with the possibility that, by contributing to genome integrity at the tissue level after the initial repair of DNA damage, cell competition could represent a significant tumor-suppressive process in mammals [63].

## Materials and methods

1. **Fly husbandry:** Flies were raised at 25°C on standard cornmeal and molasses medium, containing the following ingredients per 1L: 18g yeast; 22g molasses; 80g malt extract; 9g agar; 65g cornmeal; 2.3g methyl para-benzoic acid; 6.35ml propionic acid. Larvae were not distinguished for their sex before dissection.
2. **DNA damage γ-radiation treatment:** Flies were grown in the standard cornmeal medium at 25°C. After egg laying was done for 24 hours, the flies were transferred to a fresh vial. Following this, the larvae were allowed to grow until the wandering late 3RD instar stage, and DNA damage was induced by treating them with γ-radiation from cesium-137 using a Shepherd irradiator. For most experiments, 1500 Rads were administered, and dissection was performed at a specified time point after the irradiation.
3. **Cell death quantification:** Cell death was quantified by counting the number of Dcp-1 positive cells with the help of the ImageJ cell counter plugin. For UAS-Gal4 experiments, Dcp-1 positive cells in Gal4 to the control compartment were counted and compared as the ratio across different genetic perturbations. In the case of mutant wing discs, the number of Dcp-1 positive cells was counted in the entire disc, and the absolute number was compared.
4. **Immunohistochemistry:** Imaginal discs were fixed using 4% formaldehyde in 1 × PBS, followed by 2 h blocking with 0.5% bovine serum albumin (BSA) dissolved in 0.1% PBST (1 × PBS with 0.1% Triton X-100). Primary antibody incubations were performed at 4°C overnight, followed by 2 hours of secondary incubation at room temperature. All washes following antibody incubation were performed in 0.1% PBST. Primary antibodies were against rabbit anti-cleaved Dcp-1 (Cell Signaling 9578, 1:200), mouse anti-γH2Av (DSHB UNC93-5.2.1, 1:50), mouse anti-βGalactosidase (DSHB 40a-1, 1:50), mouse anti-p53 (DSHB 25F4, 1:50), and mouse anti-GFP (DSHB 12A6, 1:50), mouse anti-HA (CST 2367, 1:100), and anti-Xrp1 (1:250; gift from Rio lab). Alexa Fluor (Thermo Fisher-Invitrogen) and Cyanine dye conjugated secondary antibodies (Jackson Immunoresearch) were used at a 1:200 dilution. Image acquisition was performed on the Leica-Sp8 microscope, intensity adjustments were made using Photoshop CC 2018, and Image J was used for image processing.

Previous experiments have supported that significant results could be obtained from 5 replicates; in most cases, more than 5 samples were analyzed. Experiments were repeated at least 2 times or more for most of the results. No exclusion of the data points was made while performing statistical significance. For the quantitative data, N values are indicated in the figure legends. Statistical significance was performed using a Student’s t-test for independent or dependent means, as shown in the figure legends. For specific data sets, two-tailed Welch’s t-test was used, and one-way ANOVA with Tukey’s multiple comparisons test was used, as mentioned in the figure legends. Exact p-values are indicated in the figures.

### q-RT PCR

Total RNA was extracted from wing imaginal discs of late-third instar larvae treated with 2000 Rads of IR; untreated larvae were taken as a control for normalization. Wing discs were collected in TRIzol reagents (Ambion), and RNA was isolated according to the manufacturer’s instructions. RNA samples were DNase-treated according to the manufacturer’s instructions (Thermo Fisher; AM1907) and reverse transcribed using the Verso cDNA Synthesis Kit (Thermo Fisher; AB1453). The cDNA was used to perform the q-RT PCR reactions following the manufacturer’s instructions for the TaqMan Fast Advanced Master Mix (Thermo Fisher; 4444557). Custom design FAM-MGB TaqMan probes were utilized for q-RT-PCR. The gene expression values were normalized to the housekeeping gene RpL32. Similar results were obtained normalizing to actin. The following calculations were used for estimating fold change in gene expression ∆: Ct = Ct (gene of interest) – Ct (housekeeping gene), ∆∆Ct = ∆Ct (3–4 hr IR) – ∆Ct (untreated average), and Fold gene expression = 2^ -(∆∆Ct). 3 biological replicates were analysed for each condition and genotype. The gene expression analysis was performed using technical triplicates for each sample. It has been previously established that significant results can obtained 3 biological and 3 technical triplicates.

The following TaqMan probes were used in this study:

p53 Dm02154335_g1
Xrp1 Dm02142088_g1
rpr Dm01823054_s1
hid Dm01823031_m1
GstD1 Dm02362792_s1
eiger Dm01794373_m1
rad50 Dm01817359_g1
PolZ1 Dm01843262_g1
DNAlig4 Dm01838632_g1
10. Ku80 Dm01791676_g1
*Drosophila* RpL32 Dm02151827_g1
*Drosophila* actin Dm02361909_s1

## Supporting information

S1 Fig Xrp1 RNA expression in response to DNA damage. qRT-PCR analysis for Xrp1 mRNA expression levels from 3rd instar wing discs from the genotypes *w*^*1118*^, *p53*^*[5A-1-4]*^, and *Xrp1*^*m2-73*^*/Df1* collected 3–4 hours after irradiation (1500 RADs) and normalized to untreated (0 RADs) samples of the respective genotypes (A). 3RD instar wing discs assayed for *Xrp1*^*02515*^-lacZ enhancer trap expression by anti-βgal antibody in 0 Rads for *en > Xrp1 RNAi* (B), *en>Irbp18 RNAi* (C), and 4 hours after 1500 Rads treatment in genotypes *en > Xrp1 RNAi* (D), *en>Irbp18 RNAi* (E). Number of wing discs analyzed for genotypes was N = 3 for B, N = 2 for C, N = 3 for D, and N = 5 for E. Data are presented as mean ± SEM, two-way ANOVA with Tukey’s multiple comparisons test was used. P < 0.05 was considered statistically significant. Genotype: (A) *w*^*1118*^, *yw; p53*^*[5A-1-4]*^*,* and *Xrp1*^*m2-73*^*/ Df1*, (B & C) *en-Gal4::UAS-RFP, UAS-Xrp1 RNAi* (VDRC 107860); *P{PZ}Xrp1*^*02515*^, (C & E) *en-Gal4::UAS-RFP, UAS-Irbp18 RNAi*; *P{PZ}Xrp1*^*02515*^
(TIF)

S2 Fig *hid*^*20-10*^*lacZ* and *rpr*^*150*^*lacZ*intensity quantification. *hid*^*20-10*^*-lacZ* expression measured by anti-βgal staining in the wild type (no IR) (A) and 4 hours after 2000 Rads in wild type wing disc (B), *UAS-p53*^*H159N*^ (C), *UAS-Xrp1* (D), and *UAS-Irbp18* RNAi (E). (F) Mean intensity quantification for *hid*^*20-10*^*lacZ* from the wing, the anterior compartment of the wing pouch, with and without IR for the genotypes shown in S2A-E Fig. (G) The mean posterior (P) to mean anterior (A) *hid*^*20-10*^*lacZ,* with and without IR for the genotypes shown in S2A-E Fig. (H) Mean intensity quantification for *rpr*^*150*^*lacZ* from the wing, the anterior compartment of the wing pouch, with and without IR for the genotypes shown in Fig 3G-3K. (I) The mean posterior (P) to mean anterior (A) *rpr*^*150*^*lacZ* expression ratio, with and without IR for the genotypes shown in Fig 3G-3K. Data are presented as mean ± SEM, one-way ANOVA with Tukey’s multiple comparisons test was used. P < 0.05 was considered statistically significant. Genotype: (A-E & F-G) *yw*; *hid*^*20-10*^*-lacZ*, *en-Gal4::UAS-RFP, UAS-p53*^*H159N*^ (Bl. 8420)*; hid*^*20-10*^*-lacZ***,** *en-Gal4::UAS-RFP, UAS-Xrp1 RNAi* (VDRC 107860)*; hid*^*20-10*^*-lacZ***,** *en-Gal4::UAS-RFP, UAS-Irbp18 RNAi; hid*^*20-10*^*-lacZ***.** (H & I) *rpr*^*150*^-lacZ**,** *rpr*^*150*^*-lacZ, UAS-p53*^*H159N*^ (Bl. 8420)*; hh-Gal4::UAS-GFP***,** *rpr*^*150*^*-lacZ, UAS-Xrp1 RNAi* (VDRC 107860)*; hh-Gal4::UAS-GFP, rpr*^*150*^*-lacZ, UAS-Irbp18 RNAi; hh-Gal4::UAS-GFP*
(TIF)

S3 Fig Xrp1 is sufficient for the expression of p53- dependent DDR target genes. Xrp1-HA protein measured by anti-HA (red) and cell death by Dcp-1 (green) in the 3RD instar wing disc expressing UAS-RFP control (A) and UAS-Xrp1 long isoform (B) under the control of *nub-Gal4* (blue). *rpr*^*150*^*-lacZ* reporter expression measured by anti-βgal (green) and cell death by anti-Dcp-1 (red) in 3RD instar wing imaginal disc expressing UAS-RFP (C) and UAS-Xrp1 (D) under the control of *nub-Gal4*. *TRE-EGFP* reporter expression measured by anti-GFP (green) and cell death by anti-Dcp-1 (red) in 3RD instar wing imaginal disc expressing UAS-RFP control (E) and UAS-Xrp1 (F) under the control of *nub-Gal4* (blue). N = 7–8 discs were analyzed for every genotype, and each experiment was repeated at least 2 times. All the control and experimental genotypes shown in this figure were kept at 18°C to lessen the effect of Xrp1 overexpression. Genotype: (A) *nub-Gal4::UAS-RFP,* (B) *nub-Gal4::UAS-RFP::UAS-Xrp1-HA,* (C) *nub-Gal4::UAS-RFP, rpr*^*150*^*-lacZ,* (D) *nub-Gal4::UAS-RFP::UAS-Xrp1-HA, rpr*^*150*^*-lacZ,* (E) *nub-Gal4::UAS-RFP, TRE-GFP,* (F) *nub-Gal4::UAS-RFP::UAS-Xrp1-HA, TRE-GFP.*
(TIF)

S4 Fig Xrp1 contributes to the p53 O/E phenotype. (A) Control *nub-Gal4* exhibits few Dcp-1 positive cells, and no p53 protein was detected by anti-p53 antibody. (B) *nub-Gal4* driving p53 overexpression (*GUS-p53*) in the wing pouch exhibits elevated cell death measured by Dcp-1 and p53 protein. (C) Co-expressing *UAS-Xrp1* RNAi and *GUS-p53* under *nub-Gal4* led to a decrease in cell death by approximately 50%. (D) Removing one copy by *Xrp1*^*m2-73*^*/ +* did not affect cell death. (E) Removing both copies of Xrp1 (*Xrp1*^*m2-73*^*/Xrp1*^*attp flox*^) results in a decrease in cell death of approximately 50%. Xrp1-HA expression was assayed by an anti-HA antibody in the wing disc, expressing p53 under the control of *nub-Gal4* (F & F’). (G) Dcp1 intensity was quantified using ImageJ; N = 6 for each genotype. (H) Bright field photograph of *Drosophila* eye, rough eye phenotype due to constitutive expression of p53 under the control of GMR-promoter (*GMR-p53*). (I) Overexpression of dominant-negative p53 (*UAS-p53*^*H159N*^) rescues the rough eye phenotype. (J) Xrp1 knockdown by RNAi has a slight effect on the rough eye phenotype. Data are presented as mean ± SEM, one-way ANOVA with Tukey’s multiple comparisons test was used. P < 0.05 was considered statistically significant. All the control and experimental genotypes shown in this figure were kept at 18°C to lessen the effect of p53 overexpression. Genotype: (A) *nub-Gal4; w*^*1118*^, (B) *nub-Gal4, GUS-p53,* (C) *nub-Gal4, GUS-p53; UAS-Xrp1 RNAi* (Bl. 34521)*,* (D) *nub-Gal4, GUS-p53; Xrp1*^*m2-73*^*/ + ,* (E) *nub-Gal4, GUS-p53; Xrp1*^*m2-73*^*/Xrp1*^*attp flox*^*,* (F) *nub-Gal4, GUS-p53; Xrp1-HA,* (H) *GMR-p53,* (I) *GUS-p53, GMR-Gal4; UAS-p53*^*H159N*^ (Bl. 4021)*,* (J) *GUS-p53, GMR-Gal4; UAS-Xrp1 RNAi* (Bl. 4521)
(TIF)

S5 Fig Transition to p53-independent DDR occurs around 16 hours post-irradiation. (A-B) Cell death measured by anti-Dcp-1 (green and grayscale) in wing imaginal discs dissected at 2 hours after 1500 Rads, measured in en-Gal4 (posterior compartment (red)) versus anterior compartment *UAS-RFP; rpS12*^*G97D*^ (A), and *UAS-Xrp1 RNAi; rpS12*^*G97D*^ (B). Dcp-1 positive cells were quantified in anterior (A) and posterior (P) compartments and plotted as the P/A ratio (C). N = 6 discs for (A-C), data is plotted as mean ± SEM, and a student t-test, two-tailed for the independent sample, was performed for statistical significance. (D) Cell death measured by anti-Dcp-1 and damaged DNA measured by anti-γH2Av in wing imaginal discs dissected at 24 hours after 2000 Rads, measured in en-Gal4 (posterior compartment expressing Xrp1 RNAi versus anterior compartment control. The disc epithelium was projected from the apical surface to the bottom of the nuclear layer. More basal regions were not included because the vast amount of cell death that occurs in the 24h following irradiation is not all promptly cleared and accumulates in basal disc regions. (E) Quantitative comparison of P and A cell death counts, normalized to compartment area. Paired 2-tailed t-test indicated P = 0.02. (F) Quantitative comparison of P and A mean γH2Av level. Paired 2-tailed t-test indicated P = 0.0003*.* (G-K) Xrp1-HA expression in 3RD instar wing imaginal discs, no IR control disc (G), 16 hours after 1500 rads (H), *UAS-p53*^*H159N*^ (I), *UAS-Xrp1* RNAi (J), and *UAS-Irbp1* RNAi (K). 3RD instar wing imaginal discs dissected at 16 hours after 1500 Rads, cell death is measured by Dcp-1 (green and grayscale) in different genetic perturbations using en-Gal4 (posterior compartment (red)), UAS-RFP (L), *UAS-p53*^*H159N*^ (M), and *UAS-Xrp1* RNAi (N). Dcp-1 positive cells were quantified and plotted as the ratio of Dcp-1 positive cells in posterior (P) to anterior (A) compartments (O). N = 4 for (D-F) student t-test was performed to test the significance between anterior and posterior compartment. N = 10 for (L-O), data are presented as mean ± SEM, one-way ANOVA with Tukey’s multiple comparisons test was used. P < 0.05 was considered statistically significant. Genotype: (A & B) *Xrp1-HA*, (C) *en-Gal4::UAS-RFP; UAS-Xrp1 RNAi* (VDRC 107860)*; Xrp1-HA*, (D) *en-Gal4::UAS-RFP, UAS-Xrp1 RNAi* (Bl. 34521), (G) *en-Gal4::UAS-RFP; UAS-Irbp18 RNAi; Xrp1-HA*, (H) *en-Gal4::UAS-RFP; UAS-p53DN*^*H159N*^ (Bl. 8420)*; Xrp1-HA*, (I) *en-Gal4::UAS-RFP; UAS-p53DN*^*H159N*^ (Bl. 8420)*; Xrp1-HA-rpS12*^*G97D/G97D*^ (J) *en-Gal4::UAS-RFP; rpS12*^*G97D/G97D*^, (K) *en-Gal4::UAS-RFP, UAS-Xrp1 RNAi* (VDRC 107860)*; rpS12*^*G97D/G97D*^, (L) *en-Gal4::UAS-RFP, UAS-p53DN*^*H159N*^ (Bl. 8420), (M) *en-Gal4::UAS-RFP, UAS-Xrp1 RNAi* (Bl. 34521), (N) *en-Gal4::UAS-RFP, UAS-Irbp18 RNAi*
(TIF)

S1 Table Key resources table [9,49,50,107–115]. (DOCX)
