# Supplementary material for: Dual contributions of Xrp1 to genome integrity through the DNA damage response and cell competition
Source: PLoS Genet. 2026 Sep 18;22(9):e1012309. doi: 10.1371/journal.pgen.1012309 (PMC13588501; doi:10.1371/journal.pgen.1012309)
Supplement: Supplementary file 1 [file pgen.1012309.s006.docx]

**S1 Table:** Key Resources Table

| **Key Resources Table** | | | | |
| --- | --- | --- | --- | --- |
| **Reagent type (species) or resource** | **Designation** | **Source or reference** | **Identifiers** | **Additional information** |
| gene (*Drosophila melanogaster*) | Xrp1 |  | FLYBASE: FBgn0261113 |  |
| gene (*Drosophila melanogaster*) | irbp18 |  | FLYBASE: FBgn0036126 |  |
| genetic reagent (*D. melanogaster*) | p53 |  | FLYBASE: FBgn0039044 |  |
| genetic reagent (*D. melanogaster*) | rpS12 |  | FLYBASE: FBgn0286213 |  |
| genetic reagent (*D. melanogaster*) | *Xrp1^m2-73^* | (Lee et al., 2016) [9] | FLYBASE: FBal0346068 | Bloomington Drosophila Stock Center #81270 |
| genetic reagent (*D. melanogaster*) | *Xrp1^02515^* | (Spradling et al., 1999) [107] | FLYBASE: FBal0009448 | Bloomington Drosophila Stock Center #11569 |
| genetic reagent (*D. melanogaster*) | *Xrp1^attP^ ^flox^* | (Blanco et al., 2020) [49] |  | Strain maintained in Dr. Nicholas Baker’s lab |
| genetic reagent (*D. melanogaster*) | Xrp1^HA^ | (Blanco et al., 2020) [49] |  | Strain maintained in Dr. Nicholas Baker’s lab |
| genetic reagent (*D. melanogaster*) | UAS-Xrp1  (long isoform) | (Tsurui-Nishimura et al., 2013) [108] | FLYBASE: FBal0284387 |  |
| genetic reagent (*D. melanogaster*) | *irbp18^05006^* | (Francis et al., 2016) [50] | FLYBASE: FBal0185260 | Exelisis Drosophila Collection  #f05006 |
| genetic reagent (*D. melanogaster*) | *rpS12^G97D^* |  | FLYBASE: FBgn0286213 | Strain maintained in Dr. Nicholas Baker’s lab |
| genetic reagent (*D. melanogaster*) | *p53^[5A-1-4]^* |  | FLYBASE: FBgn0039044 | Bloomington Drosophila Stock Center  #6815 |
| genetic reagent (*D. melanogaster*) | UAS-p53.H159N.Ex-2 (Chr. II) |  | FLYBASE: FBti0040583 | Bloomington Drosophila Stock Center #8420 |
| genetic reagent (*D. melanogaster*) | UAS-p53.H159N.Ex -3 (Chr. 3RD) |  | FLYBASE: FBti0040584 | Bloomington Drosophila Stock Center #8421 |
| genetic reagent (*D. melanogaster*) | UAS-dsRNA^Xrp1^ | (Ni et al., 2011) [109] | FLYBASE: FBti0144696 | Bloomington Drosophila Stock Center  #34521 |
| genetic reagent (*D. melanogaster*) | UAS-dsRNA^Xrp1^ |  | FLYBASE: FBst0479673 | Vienna Drosophila Resource  Center  #107860 |
| genetic reagent (*D. melanogaster*) | UAS-dsRNA^irbp18^ | (Ni et al., 2011) [109] | FLYBASE: FBti0140125 | Bloomington Drosophila Stock Center  #33652 |
| genetic reagent (*D. melanogaster*) | UAS-dsRNA^w^ | (Ni et al., 2011) [109] | FLYBASE: FBtp0064645 | Bloomington Drosophila Stock Center  #33623 |
| genetic reagent (*D. melanogaster*) | arm-LacZ | (Vincent et al., 1994) [110] | FLYBASE: FBal0040819 |  |
| genetic reagent (*D. melanogaster*) | Ubi-GFP | (Davis et al., 1995) [111] | FLYBASE: FBal0047085 |  |
| genetic reagent (*D. melanogaster*) | M(2)56F  (mutating RpS18) | Laboratory of Y. Hiromi | FLYBASE: FBal0011916 |  |
| genetic reagent (*D. melanogaster*) | hs-FLP | (Struhl & Basler, 1993) [112] | FLYBASE: FBtp0001101 |  |
| genetic reagent (*D. melanogaster*) | ey-FLP | (Newsome et al., 2000) [113] | FLYBASE: FBal0098303 |  |
| genetic reagent (*D. melanogaster*) | GMR-gal4 | (Freeman, 1996) [114] | FLYBASE: FBtp0001315 |  |
| genetic reagent (*D. melanogaster*) | P{neoFRT}42D | (Xu & Rubin, 1993) [115] | FLYBASE: FBti0141188 | Bloomington Drosophila Stock Center  #1802 |
| genetic reagent (*D. melanogaster*) | P{neoFRT}80B | (Xu & Rubin, 1993) [115] | FLYBASE: FBti0002073 | Bloomington Drosophila Stock Center  #1988 |
| genetic reagent (*D. melanogaster*) | P{neoFRT}82B | (Xu & Rubin, 1993) [115] | FLYBASE: FBti0002074 | Bloomington Drosophila Stock Center #2050, 2051 |
| Antibody | Polyclonal Rabbit anti-XRP1 (short isoform) | (Francis et al., 2016) [50] |  | 1:200 dilution |
| Antibody | Mouse anti-HA Tag monoclonal | Cell Signalling Technology | Cat #2367  RRID:AB_10691311 | 1:100 dilution |
| Antibody | Mouse anti-beta galactosidase (mAb40-1a) monoclonal | DSHB | RRID: [AB_2314509](http://antibodyregistry.org/AB_2314509) | 1:100 dilution |
| Antibody | Rabbit anti-active-Dcp1 polyclonal | Cell Signalling Technology | Cat #9578  RRID:AB_2721060 | 1:50 dilution |
| Antibody | Mouse anti-p53 (25F4) monoclonal | DSHB | RRID: [AB_579787](http://antibodyregistry.org/AB_2314509) | 1:20 dilution |
